# Psychological distress as a host-state factor in cancer immunotherapy: clinical associations, neuroendocrine mechanisms, and future trial design

**DOI:** 10.3389/fonc.2026.1899935

**Published:** 2026-10-28

**Authors:** Guanwen Lin, Zhilin Luo

**Affiliations:** 1 Department of Anesthesiology, Hainan General Hospital, Hainan Affiliated Hospital of Hainan Medical University, Haikou, China; 2 Department of Thoracic Surgery, the Third Affiliated Hospital of Chongqing Medical University, Chongqing, China

**Keywords:** immune checkpoint inhibitors, PD-1, psychological distress, psycho-oncology, β-Adrenergic signalling

## Abstract

Immune checkpoint inhibitors (ICIs) targeting programmed cell death protein 1 (PD-1), programmed death-ligand 1 (PD-L1), and cytotoxic T-lymphocyte-associated protein 4 (CTLA-4) have transformed the treatment of multiple solid tumours, yet durable benefit remains confined to a subset of patients. Tumour-intrinsic biomarkers explain only part of this variability, highlighting the need to consider host-level determinants of antitumour immunity. Psychological distress, including depressive and anxiety symptoms, is common in patients with cancer and is potentially modifiable. In this review, we appraise clinical evidence linking pretreatment distress with ICI outcomes, with the principal clinical evidence coming from the prospective STRESS-LUNG cohort, the PRADO melanoma *post-hoc* analysis, and an individual patient-data meta-analysis of atezolizumab trials. Collectively, the available clinical evidence more strongly supports a general prognostic association, while an ICI-specific predictive effect has not been demonstrated. We then examine mechanistic evidence linking experimental chronic stress and stress-responsive signalling to sympathetic β-adrenergic and hypothalamic–pituitary–adrenal-axis pathways. These pathways can affect CD8^+^ T cells, dendritic cells, tumour cells, and the tumour microenvironment in preclinical and translational models. We also discuss psychosocial intervention, β-adrenergic blockade, and serotonergic modulation. For each approach, clinical evidence remains more limited than the mechanistic rationale. Existing evidence supports psychological distress as a clinically important and potentially modifiable host factor associated with inferior outcomes after checkpoint blockade, but does not establish that distress reduction improves ICI efficacy. Randomized, biomarker-informed studies are needed to determine whether modifying distress-related pathways can alter ICI outcomes, rather than simply identify patients with poorer prognosis.

## 1 Introduction

The introduction of immune checkpoint inhibitors (ICIs) has reshaped the treatment of advanced malignancy. By interrupting inhibitory signalling through programmed cell death protein 1 (PD-1), programmed death-ligand 1 (PD-L1), and cytotoxic T-lymphocyte-associated protein 4 (CTLA-4), these agents reinvigorate exhausted T cells and, in a subset of patients with non-small-cell lung cancer (NSCLC), melanoma, renal cell carcinoma, and other tumours, produce durable disease control that was seldom achieved with cytotoxic therapy alone. Yet durable benefit remains the exception rather than the rule, and the predictive biomarkers in routine use—PD-L1 immunohistochemistry, tumour mutational burden, and microsatellite instability—account for only a fraction of the variation in response (1). Primary, adaptive, and acquired mechanisms of resistance have been mapped in increasing detail (2), but they are overwhelmingly tumour- and microenvironment-intrinsic. Established resistance states include impaired antigen presentation, altered interferon signalling, T-cell exclusion or dysfunction, and the accumulation of regulatory T cells and suppressive myeloid populations (1, 2). Stress-responsive pathways may intersect with several of these processes. We therefore consider neuroendocrine signalling as a potential upstream host modifier of established resistance states, rather than as a separate form of ICI resistance. The persistent gap between predicted and observed benefit has prompted a complementary question: which host-level factors, extrinsic to the tumour, shape the immune response that ICIs seek to amplify?

Among candidate host factors, the neuroendocrine state of the patient has a long but, until recently, peripheral history in oncology. Negative emotional states—depression, anxiety, and chronic stress—are common in people with cancer. A meta-analysis of 24 interview-based studies placed the prevalence of major depression in oncological and haematological settings at around one in six, with some combination of mood disorder affecting 30–40% of patients in hospital settings (3); subsequent syntheses, using both interviews and validated self-report instruments, have reached broadly concordant estimates (4, 5), and prevalence varies systematically by tumour type, sex, and age (5). These states are not merely a quality-of-life (QoL) concern. Depression has been associated with increased cancer mortality across tumour types in successive meta-analyses (6–8), through mechanisms that are plausibly both biological—persistent systemic inflammation, hypothalamic–pituitary–adrenal (HPA) axis dysregulation, and impaired cellular immunity—and behavioural—delayed presentation, reduced adherence, and adverse health behaviours (6). Here, biobehavioural refers to pathways through which psychological and behavioural states influence physiological processes relevant to tumour biology. The conceptual framework linking biobehavioural processes to tumour biology through neuroendocrine signalling was articulated nearly two decades ago (9, 10) and has since been substantially extended at the level of receptor signalling and gene expression (11). What remained largely untested, until the past few years, was whether these same processes are associated with the efficacy of immunotherapy specifically—a treatment whose efficacy depends on systemic and intratumoural host immune activity rather than on tumour-intrinsic features alone, and which might therefore be unusually sensitive to host neuroendocrine state.

A distinction between clinical distress and experimental stress is important. In this review, psychological distress is used as an umbrella clinical term for depressive symptoms, anxiety symptoms, and broader emotional distress measured in patients. The PHQ-9 and GAD-7 assess depressive and anxiety symptoms, respectively; they do not directly measure chronic biological stress. Other studies use broader emotional-functioning scales or clinical diagnoses, whereas most mechanistic studies use experimental stress paradigms in animals. We therefore treat clinical distress, neuroendocrine activation, and experimental chronic stress as related but distinct levels of evidence. These terms are defined in Box 1. A recent integrative review has summarized links among chronic stress, inflammation, immune regulation, and cancer, providing broader context for the pathways considered here (12). A recent review has similarly considered psychological distress as a putative host-state determinant of immunotherapy response (13). The present review places particular emphasis on evidence hierarchy, the prognostic-versus-predictive distinction, and receptor-specific adrenergic signalling.

This review addresses four linked questions: whether pretreatment psychological distress is associated with outcomes after immune checkpoint blockade; how chronic stress may affect antitumour immunity through sympathetic β-adrenergic and HPA-axis glucocorticoid signalling; what translational strategies might follow; and how the field should distinguish prognostic association from causal, ICI-specific effects. We therefore frame psychological distress not simply as a psychosocial outcome, but as a measurable, dynamic, and potentially modifiable host-state variable that may intersect with tumour immunity, immune resistance, and immunotherapy response. Its clinical value will depend on whether future studies can integrate distress trajectories with neuroendocrine and immune biomarkers, and determine whether distress is merely a marker of disease burden or a therapeutically actionable contributor to immunotherapy resistance. The working hypothesis of this review is not that psychological distress represents an independent mechanism of ICI resistance, but that stress-related host signalling may reinforce immune states that are already known to limit checkpoint blockade.

## 2 Psychological distress in patients with cancer

### 2.1 Burden, measurement, and recognition

The epidemiological case rests on three observations. First, distress is common: interview-based and self-report syntheses converge on a major-depression prevalence near 15% and clinically significant anxiety of similar magnitude, with mixed mood disturbance considerably more frequent (3–5). Second, it is measurable with brief, validated instruments. The PHQ-9 (14) and the GAD-7 (15) are widely used measures of depressive and anxiety symptoms and were used in several recent ICI cohorts discussed below. Other studies used broader measures of emotional functioning, such as the EORTC emotional-functioning scale, and others still relied on recorded clinical diagnoses. Third, despite this, distress is under-recognized and under-treated in routine oncology—a recognition gap that is consequential if, as the following sections argue, distress is prognostically and perhaps mechanistically relevant to treatment outcome. Heterogeneity in the instrument used and in its threshold is one reason that effect estimates across studies are not directly comparable, a point we return to below. Study-specific thresholds are screening definitions and should not be interpreted as validated prognostic cut-points for ICI outcome. The exact instrument, threshold, and timing used in each clinical study are reported in **Table 1**.

**Table 1:** Clinical studies relating psychological or emotional distress to outcomes in patients receiving immune checkpoint inhibitors, with comparator data where available.

Study (first author, year, PMID)	Setting: tumour type and stage	Design / N / exposure groups / follow-up	Psychological measure, threshold, timing	Treatment regimen	Endpoint(s)	Covariates adjusted	Effect estimate (95% CI)	Formal treatment-by-distress interaction reported?	Key limitation (factual only)
Derry-Vick et al., 2025 (16) PMID: 41176702	Multiple advanced cancers; lung cancer predominant	Multicentre retrospective chart-based cohort; N=913. Anxiety diagnosis: 102; depression diagnosis: 106; both: 41. Follow-up through Jan 2021.	Pre-existing chart-recorded anxiety and/or depression diagnosis before ICI; no symptom-scale threshold.	Anti-PD-1/PD-L1 monotherapy: nivolumab (58%), pembrolizumab (35%), atezolizumab (5%).	Any-grade irAEs; OS; TTF	Age, sex, race, plus endpoint-specific clinical covariates selected by univariable screening.	Anxiety: OS HR 0.74 (0.54–1.00), p=0.048; any-grade irAEs OR 1.80 (1.16–2.79), p=0.009. Depression was non-significant for irAEs, OS, and TTF in univariable analyses and was not entered into multivariable models.	No formal interaction reported; no treatment comparator.	Retrospective chart diagnosis does not measure current symptom burden; psychiatric treatment/medication data unavailable.
Zhu et al., 2026 (17) PMID: 40851222	Stage IV NSCLC	Retrospective cohort; N=80. HADS-A >7: 8/80; HADS-D >7: 21/80. Follow-up duration not separately reported.	Baseline HADS. HADS-A >7 and HADS-D >7 were used for categorical survival analyses; HADS-A and HADS-D scores were entered as continuous variables in the Cox models.	ICI monotherapy. First line: pembrolizumab, atezolizumab, or tislelizumab (35/80). Second line: atezolizumab, tislelizumab, or pembrolizumab (45/80).	PFS; OS; week-8 tumour response	Variables significant in univariable screening, including CONUT, HHI, HADS-A/HADS-D, BFI, and AIS; disease progression also entered the OS model.	Multivariable PFS: HADS-A, per 1-point increase, HR 1.122 (1.023–1.230), p=0.015. Multivariable OS: HADS-D, per 1-point increase, HR 1.094 (1.015–1.180), p=0.019.	No formal interaction reported; single-treatment-class cohort.	Retrospective, small cohort; no treatment comparator; anxiety and depression were analysed separately rather than as one ED construct.
Zeng et al., 2024 (STRESS-LUNG) (18) PMID: 38740994	Advanced unresectable/recurrent NSCLC (stage IIIB–IV)	Prospective observational cohort; N=227 (ED 111; no ED 116). Biomarker subset N=210. Data cut-off: 30 Nov 2023.	Pretreatment PHQ-9 ≥5 and/or GAD-7 ≥5.	First-line PD-1/PD-L1 blockade, alone or with chemotherapy; 94.7% received combination ICI-based treatment.	PFS (primary); ORR; OS; QoL; exploratory cortisol/ACTH	Sensitivity analyses used PSM/IPTW for baseline imbalance; sex and pathology were included because of association with ED.	PFS HR 1.73 (1.23–2.43); ORR OR 0.54 (0.32–0.91); 2-year OS HR 1.82 (1.12–2.97). Biomarker subset: high vs low cortisol PFS HR 1.55 (1.09–2.19), OS HR 1.82 (1.10–3.02).	No formal treatment-by-distress interaction reported.	Observational design; residual confounding remains possible; most patients received combination regimens; cortisol analysis was observational/exploratory.
Fraterman et al., 2023 (PRADO post-hoc) (19) PMID: 37957378	Stage IIIB–D melanoma	Post-hoc analysis of phase 2 PRADO; N=88 (ED 28; no ED 60). Two-year RFS and DMFS reported.	Pretreatment EORTC QLQ-C30 emotional-functioning score ≤71 = ED; >71 = no ED.	Neoadjuvant ipilimumab + nivolumab.	MPR; 2-year RFS; 2-year DMFS	MPR model adjusted for IFN-γ signature and TMB; survival models adjusted for IFN-γ signature.	MPR: adjusted OR 0.20, p=0.038; 2-year RFS: adjusted HR 3.81, p=0.034; 2-year DMFS: adjusted HR 4.33, p=0.040. 95% CI not reported in source for these adjusted estimates.	No formal treatment-by-distress interaction reported; single treatment regimen.	Post-hoc analysis; modest subgroup size; trial was not designed to test a distress-by-treatment interaction.
Li et al., 2025 (IPD meta-analysis) (20) PMID: 40970247	Advanced NSCLC across seven atezolizumab trials	IPD meta-analysis; N=4,632 (ED 1,802; no ED 2,830); ICI 3,162 and chemotherapy 1,470. Follow-up varied across trials.	Baseline EORTC QLQ-C30 emotional-functioning standard score ≤71 = ED; >71 = no ED.	Atezolizumab-containing regimens (including combination regimens) and chemotherapy comparator arms across seven trials.	OS; PFS	Multivariable models adjusted for age, sex, BMI, tumour stage, and metastatic burden; cohort-level estimates combined across trials.	Overall: OS HR 1.18 (1.07–1.30); PFS HR 1.15 (1.03–1.28). OS subgroup: ICI HR 1.18 (1.04–1.34); chemotherapy HR 1.18 (1.00–1.39).	Not reported. Similar subgroup HRs do not substitute for a formal treatment-by-distress interaction test.	Treatment regimens and trials were heterogeneous; authors classified ED as prognostic rather than ICI-specific predictive.
Lin et al., 2026 (aggregate meta-analysis) (21) PMID: 42245743	Advanced NSCLC	Systematic review/meta-analysis; 8 studies, N=911. Two ICI-based studies, two chemotherapy-only studies, four mixed-treatment studies. Follow-up varied.	Study-specific emotional-distress definitions; measures and thresholds varied across included studies.	Heterogeneous: ICI-based therapy ± chemotherapy, chemotherapy alone, or mixed regimens depending on study.	OS; PFS; ORR	No common patient-level covariate set; pooled study-level effect estimates from included studies.	Pooled OS HR 1.85 (1.50–2.28); PFS HR 1.80 (1.22–2.66); ORR OR 0.55 (0.37–0.80).	No patient-level interaction. Study-level subgroup-difference tests only: OS p=0.94; PFS p=0.14; ORR p=0.76.	Only two included studies were ICI-based; heterogeneous distress measures and regimens; certainty rated low for OS/ORR and very low for PFS.
Tian et al., 2025 (22) PMID: 40786522	Advanced gastric cancer	Prospective cohort with PSM; N=104 (ED 46; no ED 58). Median follow-up 19.7 months.	PHQ-9 ≥5 and/or GAD-7 ≥5 within 1 week before ICI treatment.	Anti-PD-1-based combination therapy: ICI + chemotherapy (67.3%), ICI + targeted therapy (21.2%), or ICI + chemotherapy + targeted therapy (11.5%).	OS; PFS; ORR; DCR	Multivariable Cox models adjusted for ECOG PS, TNM stage, and treatment line; PSM used baseline clinical covariates selected in the source analysis.	OS HR 2.04 (1.27–3.25); PFS HR 3.01 (1.92–4.70); DCR RR 0.50 (0.34–0.74).	No formal treatment-by-distress interaction reported; no randomized treatment comparator.	Modest cohort; heterogeneous combination regimens; PSM cannot eliminate residual confounding.
Huang et al., 2026 (23) PMID: 41588106	Advanced, treatment-naïve, inoperable gastro-oesophageal cancer	Prospective observational cohort; N=84 (ED 43; no ED 41). Median follow-up 14.2 months.	Baseline PHQ-9 + GAD-7 combined score ≥10; assessment median 3 days before ICI. Sensitivity analysis used PHQ-9 ≥5 and/or GAD-7 ≥5.	First-line sintilimab, tislelizumab, camrelizumab, toripalimab, or pembrolizumab; monotherapy or, predominantly, with chemotherapy (88.1%).	PFS (primary); DCR (secondary); exploratory peripheral biomarkers	Multivariable Cox/logistic models included baseline sociodemographic and clinical variables; final PFS model retained ED and PD-L1 CPS.	PFS HR 2.59 (1.35–4.97). DCR 39.5% vs 68.3% (ED vs no ED).	No formal treatment-by-distress interaction reported.	Small cohort; mixed ICI regimens; biomarker analyses exploratory; distress definition differs from STRESS-LUNG.
Wang et al., 2026 (24) PMID: 41778154	Recurrent high-grade glioma	Prospective single-centre cohort; N=75 (ED 36; no ED 39). Median follow-up 14.9 months.	PHQ-9 ≥5 and/or GAD-7 ≥5 within 2 weeks before ICB.	Tislelizumab + bevacizumab + temozolomide.	OS; PFS; ORR; CBR	Firth multivariable model adjusted for age, KPS, IDH1 mutation, baseline steroid use, caregiver status, and ED.	OS 15.8 vs 32.3 months; unadjusted HR 2.40 (1.22–4.72), p=0.006; Firth-adjusted HR 2.74 (1.27–5.92), p=0.01. PFS 3.4 vs 7.8 months; ORR 10.7% vs 48.5%.	No formal treatment-by-distress interaction reported; no treatment comparator.	Small single-centre cohort; disease-specific combination regimen; no comparator arm; distress assessed at baseline only.

“95% CI not reported in source” indicates that the primary report did not provide the interval; no CI was inferred from a P value. “Formal treatment-by-distress interaction” refers to an explicit statistical interaction between treatment and distress; separate subgroup estimates do not substitute for this test.

AIS, Athens Insomnia Scale; BFI, Brief Fatigue Inventory; CBR, clinical benefit rate; CI, confidence interval; CONUT, Controlling Nutritional Status; CPS, combined positive score; DCR, disease control rate; DMFS, distant-metastasis-free survival; ED, emotional distress; EORTC QLQ-C30, European Organisation for Research and Treatment of Cancer Quality of Life Questionnaire-Core 30; GAD-7, Generalized Anxiety Disorder-7; HADS-A/HADS-D, Hospital Anxiety and Depression Scale anxiety/depression subscales; HHI, Herth Hope Index; ICB, immune checkpoint blockade; ICI, immune checkpoint inhibitor; irAE, immune-related adverse event; KPS, Karnofsky Performance Status; MPR, major pathological response; NSCLC, non-small-cell lung cancer; OR, odds ratio; ORR, objective response rate; OS, overall survival; PFS, progression-free survival; PHQ-9, Patient Health Questionnaire-9; PSM, propensity score matching; QoL, quality of life; RFS, relapse-free survival; TMB, tumour mutational burden; TTF, time to treatment failure.

### 2.2 Distress and oncological prognosis

That depression predicts worse cancer outcomes is among the more robust findings in psycho-oncology, demonstrated in meta-analyses that long predate immunotherapy (6–8). The proposed biological mediators are the same pathways examined in Section 3, which is what makes the immunotherapy setting a sharp test of the hypothesis (9, 11). Non-biological pathways may also contribute. Distress can coexist with lower treatment adherence, missed appointments, physical inactivity, poor nutrition, and financial or transportation difficulties. These factors may influence outcome independently of neuroendocrine signalling and can act as confounders, mediators, or consequences of more advanced disease. Disentangling these strands—and excluding reverse causation, in which more advanced disease produces distress rather than the reverse—is the defining analytic problem of the field.

### 2.3 Distress as a distinctive class of host factor

Psychological distress is clinically relevant in part because it can be addressed through psychosocial care and, when indicated, pharmacological treatment. Tumour-derived biomarkers, by contrast, are primarily used to guide treatment selection. Whether modifying distress changes ICI outcomes is a separate question that current evidence does not answer. We therefore distinguish the clinical modifiability of distress from any claim that it is a causal determinant of ICI response.

## 3 Clinical evidence linking distress to immune checkpoint inhibitor outcomes

A summary of the principal clinical studies, their designs, and their limitations is provided in **Table 1**.

Most available clinical data concern PD-1 or PD-L1 blockade, alone or in combination with other agents. Current studies are too heterogeneous to determine whether the association with distress differs by checkpoint target. The studies also differ in how the psychological exposure is defined: some use symptom scales, some use broader emotional-functioning scores, and some use recorded psychiatric diagnoses. These exposures should not be assumed to be equivalent.

A central difficulty in interpreting these studies is reverse causation and residual confounding. Greater disease burden can increase psychological symptoms while also worsening performance status, inflammation, corticosteroid exposure, sleep, physical activity, treatment adherence, and survival. Observational associations between distress and outcome therefore cannot determine whether distress is a cause, a mediator, or a marker of an adverse clinical state. **Figure 1** sets out these relationships as a conceptual framework, and the corticosteroid literature provides a concrete example of the same problem (Section 5.1).

**Figure 1:**
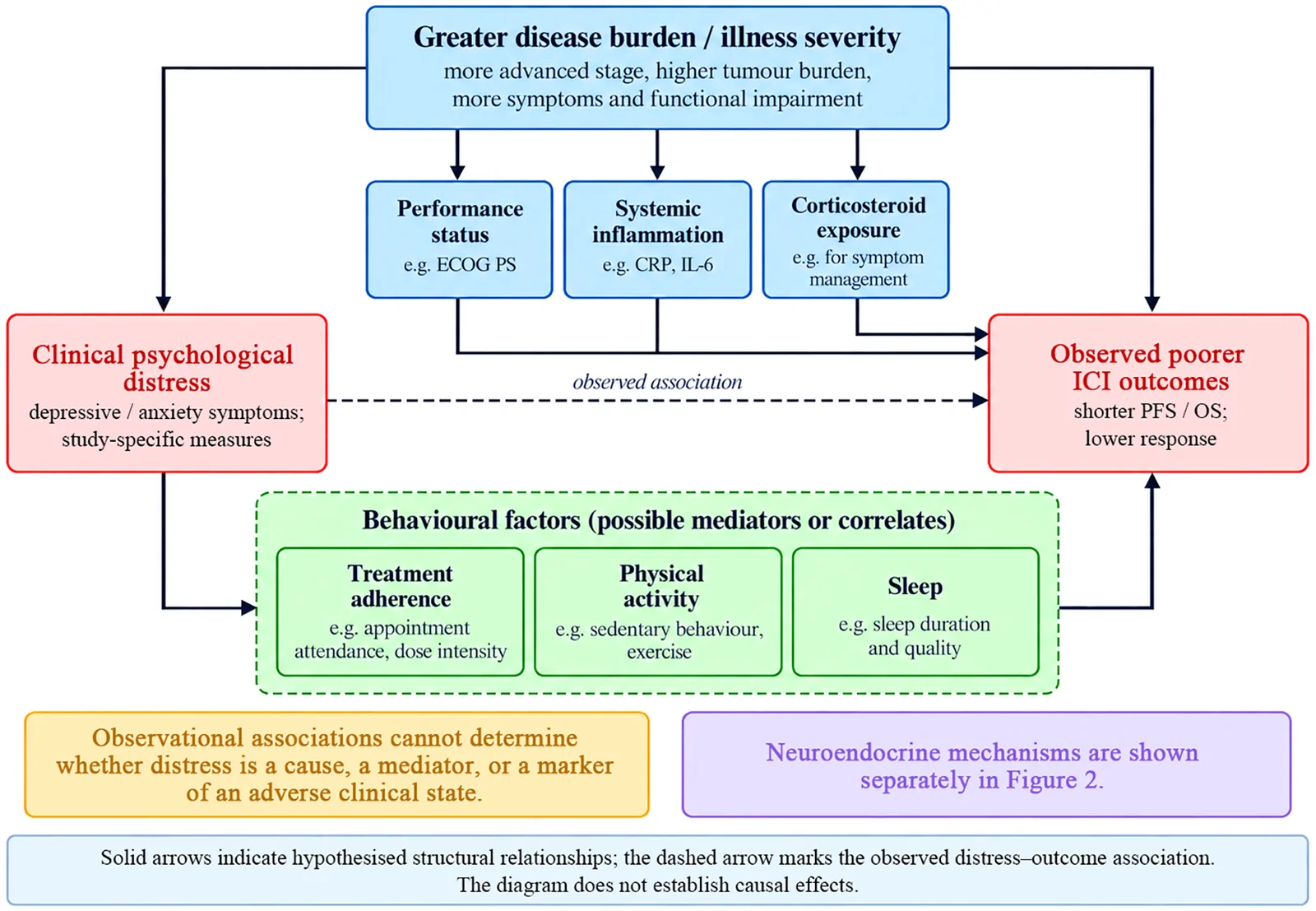
Conceptual framework for interpreting observational associations between psychological distress and immune checkpoint inhibitor outcomes. Greater disease burden may increase psychological distress while also affecting performance status, systemic inflammation, corticosteroid exposure, treatment adherence, physical activity, sleep, and survival. Behavioural factors may lie on mediating pathways, but may also correlate with disease severity and other prognostic factors. The diagram is restricted to confounding and mediation structures relevant to observational interpretation; neuroendocrine mechanisms are shown separately in **Figure 2**. Solid arrows indicate hypothesised structural relationships; the dashed arrow marks the observed distress–outcome association. The diagram does not establish causal effects.

### 3.1 Retrospective and observational signals

Retrospective evidence is heterogeneous and depends strongly on how psychological morbidity is defined. In a multicentre chart-based cohort of 913 patients receiving anti-PD-1 or anti-PD-L1 monotherapy, a pre-existing anxiety diagnosis was associated with longer overall survival (OS) after multivariable adjustment (HR 0.74, 95% CI 0.54–1.00; p=0.048) and with a higher likelihood of any-grade immune-related adverse events (OR 1.80, 95% CI 1.16–2.79; p=0.009) (16). Depression was not significantly associated with irAEs or OS in univariate analyses and therefore was not entered into the multivariable models. The authors noted that chart-recorded diagnoses do not measure current symptom burden and that psychiatric treatment and medication data were unavailable. This pattern is difficult to reconcile with a simple model of uniformly suppressed immune activity and reinforces the need to distinguish recorded diagnoses from current symptom scales. A separate retrospective cohort of 80 patients with stage IV NSCLC receiving ICI monotherapy used the Hospital Anxiety and Depression Scale and reported associations between higher anxiety and shorter PFS and between depression and shorter OS (17). Because that study was small, retrospective, and evaluated multiple baseline clinical and patient-reported factors, detailed cut-offs and effect estimates are reported in **Table 1** rather than expanded further here.

### 3.2 The STRESS-LUNG cohort: prospective observational evidence in NSCLC

The prospective STRESS-LUNG cohort enrolled 227 patients with advanced NSCLC starting first-line ICI-based therapy (18). Emotional distress was assessed before treatment and was defined as PHQ-9 ≥5 and/or GAD-7 ≥5; 111 patients (48.9%) met this definition. On the primary endpoint, patients with distress had a shorter median progression-free survival than those without (7.9 vs 15.5 months; hazard ratio [HR] 1.73, 95% CI 1.23–2.43; p=0.002). Secondary endpoints were concordant: a lower objective response rate (46.8% vs 62.1%; odds ratio 0.54, 95% CI 0.32–0.91; p=0.022) and a lower 2-year OS (46.5% vs 64.9%; HR for death 1.82, 95% CI 1.12–2.97; p=0.016), with poorer QoL scores throughout (18). Because the study was observational, these findings establish association rather than causation.

Prospective enrolment, prespecified endpoints, and standardized instruments reduce several limitations of chart-based studies. An exploratory biomarker analysis assessed baseline serum cortisol and ACTH in 210 patients. Blood sampling was performed at 08:00 before ICI treatment. Cortisol was higher in patients with distress than in those without (443.4 vs 386.0 nmol/L; p=0.019), whereas ACTH did not differ significantly (18). Using the cohort median cortisol level as the cut-off, median PFS was 7.8 months in the high-cortisol group and 12.6 months in the low-cortisol group (HR 1.55, 95% CI 1.09–2.19; p=0.014). Higher cortisol was also associated with a greater risk of OS events (HR 1.82, 95% CI 1.10–3.02; p=0.021). These findings are biologically relevant but remain observational and do not establish cortisol as a mediator between distress and ICI outcome.

### 3.3 Melanoma and convergence across tumour types

In a *post-hoc* analysis of the phase 2 PRADO trial of neoadjuvant ipilimumab plus nivolumab in stage III melanoma, baseline distress—identified with the European Organisation for Research and Treatment of Cancer (EORTC) emotional-functioning scale—was associated with a lower major pathological response rate (46% vs 65%; adjusted odds ratio 0.20; p=0.038), a lower 2-year relapse-free survival (74% vs 91%; adjusted HR 3.81; p=0.034), and a lower 2-year distant-metastasis-free survival (78% vs 95%; adjusted HR 4.33; p=0.040), each after adjustment for the interferon-γ signature and tumour mutational burden (19). However, distress was examined *post hoc* in a relatively small subgroup, and the effect estimates require confirmation.

A large clinical dataset is an individual patient-data (IPD) meta-analysis of 4632 patients across seven atezolizumab trials in NSCLC, which found that emotional distress was associated with worse OS (HR 1.18, 95% CI 1.07–1.30) and progression-free survival (HR 1.15, 95% CI 1.03–1.28), with effects evident whether patients received immunotherapy or chemotherapy (20). Emotional distress was defined using the European Organisation for Research and Treatment of Cancer Quality of Life Questionnaire-Core 30 (EORTC QLQ-C30) emotional-functioning scale: a standard score ≤71 defined ED and >71 defined non-ED. This threshold is an EORTC emotional-functioning classification rather than a psychiatric diagnostic criterion and is not directly equivalent to PHQ-9 or GAD-7 symptom thresholds. Subgroup IPD meta-analysis showed the same OS point estimate in the ICI cohort (HR 1.18, 95% CI 1.04–1.34) and the chemotherapy cohort (HR 1.18, 95% CI 1.00–1.39) (20). The authors concluded that ED was a prognostic biomarker rather than an ICI-specific predictive biomarker. However, a formal treatment-by-distress interaction test was not reported. The similar subgroup estimates and the authors’ interpretation therefore support a general prognostic association, but they do not statistically establish the absence of treatment interaction. A separate aggregate-data meta-analysis included eight studies and 911 patients with advanced NSCLC (21). Distress was associated with worse OS (HR 1.85, 95% CI 1.50–2.28), PFS (HR 1.80, 95% CI 1.22–2.66), and objective response (OR 0.55, 95% CI 0.37–0.80). Only two of the eight studies included ICI-treated patients. Study-level subgroup analyses did not identify significant differences according to treatment modality for OS (p=0.94), PFS (p=0.14), or ORR (p=0.76). The two analyses overlap in their constituent cohorts and are therefore not independent. These aggregate subgroup tests do not substitute for an individual-level treatment-by-distress interaction analysis, and the low-to-very-low GRADE certainty of the aggregate analysis further limits its independent evidentiary weight.

### 3.4 Extension to other tumour types

Beyond NSCLC and melanoma, prospective cohorts have reported associations in other tumour types. In advanced gastric cancer, Tian et al. prospectively studied 104 patients receiving anti-PD-1-based combination therapy (22). Emotional distress was defined as PHQ-9 ≥5 and/or GAD-7 ≥5 within 1 week before ICI treatment and was associated with shorter OS (HR 2.04, 95% CI 1.27–3.25) and PFS (HR 3.01, 95% CI 1.92–4.70). In advanced inoperable gastro-oesophageal cancer, Huang et al. studied 84 treatment-naïve patients (23); distress was defined as a combined PHQ-9 plus GAD-7 score ≥10 and was associated with shorter PFS (7.8 vs 14.0 months; HR 2.59, 95% CI 1.35–4.97) and a lower DCR (39.5% vs 68.3%). In recurrent high-grade glioma, Wang et al. studied 75 patients receiving tislelizumab, bevacizumab, and temozolomide (24); distress was defined as PHQ-9 ≥5 and/or GAD-7 ≥5 within 2 weeks before ICI treatment and remained associated with poorer OS in Firth-adjusted analysis (HR 2.74, 95% CI 1.27–5.92). High-grade glioma also has distinct tumour- and treatment-related immune constraints. These include systemic lymphopenia (25) and stem-cell-associated immune evasion (26). In a phase I study, long-acting IL-7 increased lymphocyte counts and expanded CD8^+^ T-cell clonotypes in patients with high-grade glioma (25). A recent conference abstract also reported that lower baseline lymphocyte counts were associated with shorter OS after pembrolizumab in recurrent glioblastoma (27). These studies extend the clinical literature beyond NSCLC and melanoma, but their modest sample sizes, tumour-specific contexts, and residual confounding limit direct comparison. Taken together, the available studies support an association between pretreatment distress and poorer outcomes in several ICI-treated cohorts, while stopping well short of demonstrating causation.

Across these clinical studies, distress was measured with symptom scales, emotional-functioning scores, or recorded diagnoses. None of these measures is equivalent to an experimental chronic-stress exposure, which is considered separately in the mechanistic section. The inconsistency in direction noted in Section 2.1 further supports keeping these constructs separate. The available cohorts also differ in age, sex distribution, performance status, line of therapy, and prior treatment exposure. Available studies were not designed to establish whether the distress-outcome association differs across these patient characteristics, which limits cross-study comparison.

## 4 Neuroendocrine mechanisms linking chronic stress to impaired antitumour immunity

Experimental chronic stress activates two neuroendocrine systems: the sympathetic nervous system, which releases catecholamines, and the HPA axis, which culminates in glucocorticoid secretion (28). Much of the mechanistic evidence comes from animal models or receptor-manipulation experiments, whereas human data mainly consist of tumour or tissue observations, circulating biomarker associations, and non-randomized clinical studies. Throughout Section 3, we identify the evidence context where it materially affects interpretation. These labels are descriptive and non-exhaustive, and they should not be read as an ordinal evidence scale. We consider them in turn, then their convergence on the tumour microenvironment and the behavioural routes that compound them (**Figure 2**). A distinction runs through the mechanistic literature and should be made explicit. One body of work shows that stress-responsive signalling can promote tumour growth, angiogenesis, invasion, and metastatic dissemination (29–31). A second body of work tests whether the same signalling alters immune effector functions or checkpoint-blockade efficacy (32–34). Only studies that directly test checkpoint response constitute evidence for an ICI-specific resistance mechanism. We therefore treat tumour-progression effects and checkpoint-response effects as related but separable.

**Figure 2:**
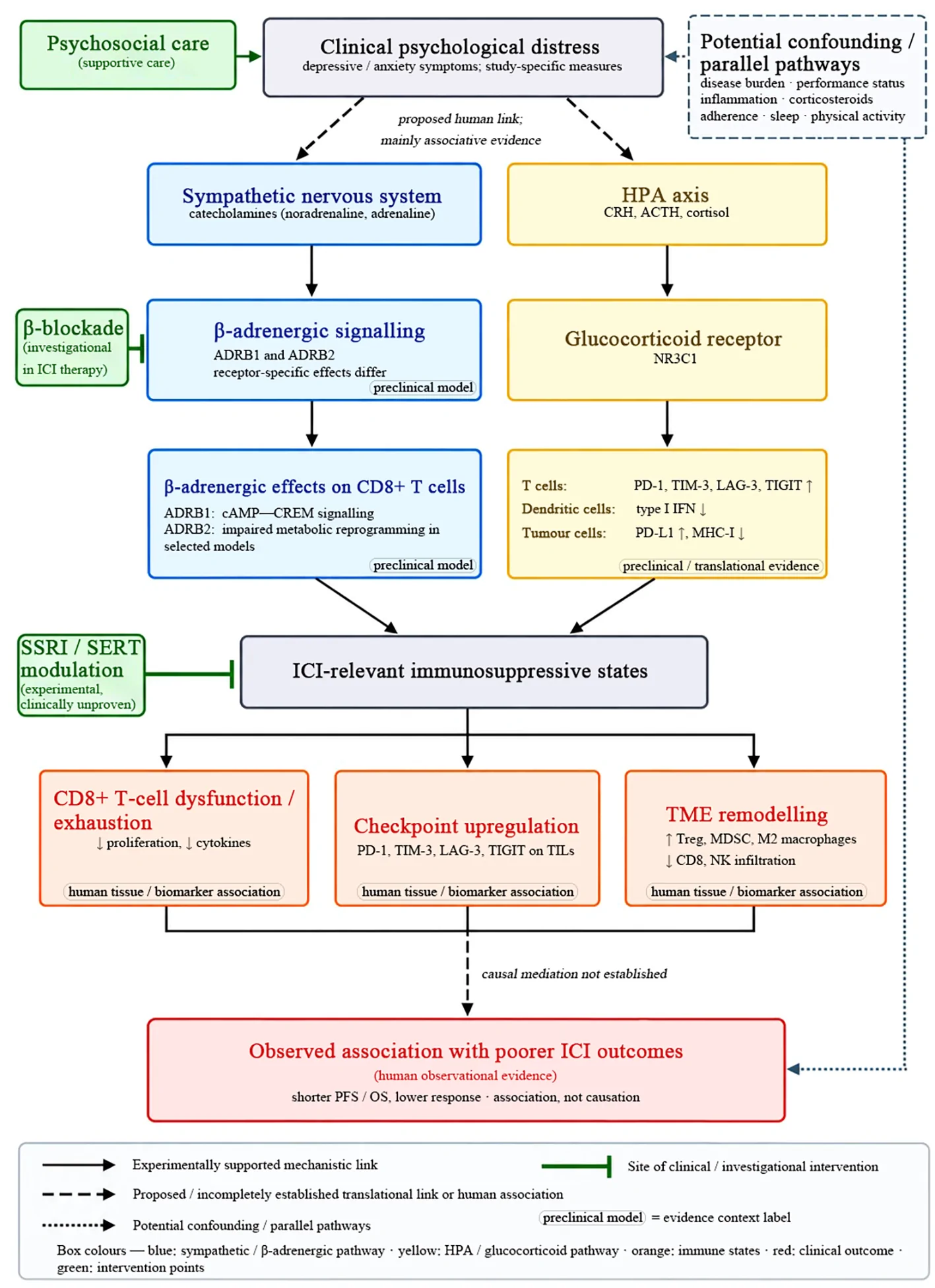
Evidence framework linking clinical psychological distress, stress-responsive neuroendocrine signalling, and immune states relevant to checkpoint blockade. Clinical distress is measured in patients using study-specific psychological instruments and should not be equated with experimental chronic stress. Experimental and translational studies show that catecholamine signalling through ADRB1 and ADRB2 can affect T-cell function, while glucocorticoid signalling through NR3C1 can influence inhibitory receptor expression in T cells, type I interferon responses in dendritic cells, and PD-L1 or MHC class I expression in tumour cells. These mechanisms overlap with recognised immunosuppressive states relevant to ICI resistance. The association between psychological distress and poorer ICI outcomes is based on human observational studies and does not establish that the mechanistic pathways shown mediate the clinical association. Evidence context is indicated directly in the figure as preclinical model, human tissue or biomarker association, or human observational evidence. Solid arrows denote experimentally supported mechanistic links; dashed arrows denote proposed or incompletely established translational links; dotted arrows indicate potential confounding or parallel pathways. Points at which the interventions discussed in Section 4 would act are superimposed.

### 4.1 Catecholamines and β-adrenergic signalling

Early studies established that sympathetic signalling can affect tumour biology. In a breast-cancer model, chronic stress accelerated metastasis through β-adrenergic recruitment of pro-tumour macrophages to the primary tumour (29), and stress promoted tumour growth and angiogenesis in an ovarian-carcinoma model (30). Sympathetic innervation itself contributes to tumour initiation and progression in prostate cancer (31), and the broader regulation of the tumour microenvironment by the sympathetic nervous system—spanning angiogenesis, inflammation, epithelial–mesenchymal transition, and the suppression of cellular immunity—has been comprehensively reviewed (11). These studies mainly addressed tumour growth and metastasis rather than ICI resistance. More direct evidence comes from studies of adrenergic signalling in CD8^+^ T cells within checkpoint-blockade models.

The mechanistic picture has been clarified substantially by work identifying the β1-adrenergic receptor (ADRB1) as a regulator of CD8^+^ T-cell exhaustion (32). Exhausted CD8^+^ T cells upregulate ADRB1; exposure of ADRB1^+^ T cells to catecholamines suppresses their cytokine production and proliferation; and within tumours, exhausted T cells cluster around sympathetic nerves in an ADRB1-dependent manner (32). Signalling proceeds through cyclic adenosine monophosphate (cAMP) and the cAMP-responsive element modulator (CREM), and forced ADRB1 expression impairs T-cell-receptor signalling while upregulating inhibitory receptors (32). Crucially, ablation of β1-adrenergic signalling—genetically or with the β1-selective blocker atenolol—limited the progression of T cells towards exhaustion and improved effector function when combined with checkpoint blockade in melanoma and colon-cancer models, and in an ICI-resistant pancreatic-cancer model a β-blocker and checkpoint blockade synergized to restore CD8^+^ T-cell responses (32).

The receptor subtype matters, and the literature is not uniform on which subtype dominates. The exhaustion mechanism above is attributed to ADRB1 (32). An independent and substantial body of work, however, implicates the β2-adrenergic receptor in adrenergic suppression of CD8^+^ T-cell metabolism and effector phenotype: chronic adrenergic stress drives metabolic dysfunction and an exhausted phenotype that is relieved by propranolol or by β2-receptor deficiency (33), housing-temperature stress undermines checkpoint-inhibitor efficacy through β2-adrenergic activation (34), and standard-temperature housing suppresses a CD8^+^ effector phenotype in a manner that compromises immunotherapy (35). The receptor subtypes are not mutually exclusive—catecholamines engage several—but the distinction is not academic: a β1-selective and a non-selective agent would be predicted to differ in immunological effect if the dominant target is ADRB1 rather than the β2-adrenergic receptor (ADRB2), and this directly conditions the interpretation of the clinical β-blocker data in Section 4. The relative contribution of ADRB1 and ADRB2 in human tumour-infiltrating T cells remains uncertain, which complicates interpretation of β1-selective and non-selective β-blockers.

In murine models, adrenergic signalling constrains the metabolic reprogramming required for CD8^+^ effector function, raises PD-1 on tumour-infiltrating lymphocytes, and reduces the efficacy of PD-1/PD-L1 blockade; relief of adrenergic tone restores intratumoral CD8^+^ frequency and function, and this benefit is lost after CD8^+^ depletion (32, 33, 35). This provides direct preclinical evidence that adrenergic signalling can modify checkpoint-blockade response. Whether the same dependency operates in human tumours has not been established.

### 4.2 The HPA axis and glucocorticoid signalling

HPA-axis activation culminates in cortisol secretion, and glucocorticoids act through the glucocorticoid receptor (encoded by NR3C1) to exert broad, long-recognized immunosuppressive effects (36). For immunotherapy, particularly relevant are studies that connect glucocorticoid signalling specifically to the checkpoint programme, rather than to nonspecific immunosuppression alone. PD-1, TIM-3, LAG-3, and TIGIT are inhibitory receptors associated with dysfunctional or exhausted T-cell states, and their co-expression marks reduced effector capacity in the tumour microenvironment. First, the glucocorticoid receptor is highly expressed by dysfunctional or exhausted tumour-infiltrating CD8^+^ T cells, where it transactivates the inhibitory receptors PD-1, T-cell immunoglobulin and mucin-domain containing-3 (TIM-3), lymphocyte-activation gene 3 (LAG-3), and T-cell immunoreceptor with immunoglobulin and ITIM domains (TIGIT), and the immunosuppressive cytokine interleukin-10 (IL-10); conditional deletion of the receptor in CD8^+^ T cells improves effector differentiation and restrains the dysfunctional phenotype, and active glucocorticoid signalling marks failure to respond to checkpoint blockade in both murine models and melanoma patients (37). This places glucocorticoid exposure directly upstream of the checkpoint molecules that ICIs target. Second, stress-induced glucocorticoids induce the glucocorticoid-responsive gene TSC22D3 (encoding GILZ) in dendritic cells, blocking type I interferon (IFN) responses and IFN-γ^+^ T-cell activation and thereby compromising therapy-induced antitumour immunity (38)—a mechanism that links psychological stress, through a defined transcriptional intermediary, to the failure of immunogenic therapy. In experimental models, the effect is reversed by glucocorticoid-receptor antagonism or by dendritic-cell-specific Tsc22d3 deletion, which establishes the gene as a required intermediary rather than a correlate (38). Third, glucocorticoid-receptor signalling has a tumour-cell-intrinsic role: in pancreatic ductal adenocarcinoma it upregulates PD-L1 and represses major histocompatibility complex (MHC) class I, and receptor inhibition reverses both, enhances cytotoxic T-cell infiltration, and overcomes resistance to checkpoint blockade (39). This is a loss-of-function result, which places the receptor upstream of the resistant phenotype rather than alongside it (39). Glucocorticoids therefore act on both sides of the immune synapse, raising inhibitory signals on T cells and on tumour cells alike. The cortisol finding in STRESS-LUNG is consistent with this biology but remains an observational biomarker association (18).

### 4.3 Crosstalk and remodelling of the tumour microenvironment

The sympathetic and HPA systems are interconnected rather than parallel: catecholamine signalling can stimulate corticotropin-releasing hormone (CRH) and adrenocorticotropic hormone (ACTH) release, while glucocorticoids feed back on sympathetic activity, producing an integrated stress response (28). Their combined effect on the tumour microenvironment is consistent across models—reduced infiltration by cytotoxic CD8^+^ T cells and natural killer cells, with accumulation of regulatory T cells, myeloid-derived suppressor cells, and M2-polarised macrophages (11, 29). Neuroendocrine effects are not confined to immune cells. Adrenergic signalling has also been linked to angiogenesis, tumour-cell invasion, and stromal changes (11, 29–31), while glucocorticoid-receptor signalling can act directly in tumour cells, including regulation of PD-L1 and MHC class I (39). These changes overlap with the recognized features of ICI resistance set out in the Introduction. The available evidence therefore supports stress signalling as a possible upstream modifier of the resistant tumour microenvironment, rather than as a distinct resistance pathway. Blocking one neuroendocrine pathway may leave other stress-responsive pathways intact. This is one possible explanation for the inconsistent clinical findings with β-blockade, although it has not been tested directly.

### 4.4 Behavioural and contextual pathways

Psychological distress also coexists with behavioural and social factors that can influence cancer outcomes without requiring a neuroendocrine mechanism. These include sleep disturbance, physical inactivity, poor nutrition, reduced treatment adherence, missed appointments, and financial or transportation difficulties. Such factors can act as mediators or confounders in observational studies of distress and survival, as shown in **Figure 1**. Experimental stress paradigms are also heterogeneous. Restraint stress, social isolation, and housing-temperature stress are not interchangeable exposures, and findings from one model should not be generalized to all forms of experimental stress. A recent melanoma model combined thermoneutral housing with circadian optimization of anti-PD-1 treatment and produced a synergistic antitumour effect in an otherwise resistant model. This supports host physiological state as a modifier of checkpoint response rather than a separate resistance mechanism (40). Adrenergic signalling is also context dependent. In murine models, exercise-induced epinephrine mobilized natural killer cells and suppressed tumour growth, and this effect was attenuated by β-adrenergic blockade (41). Acute physiological adrenergic activation should therefore not be assumed to have the same immune effects as chronic experimental stress. These behavioural pathways matter because any trial that modifies distress must contend with them, as confounders or as co-interventions.

## 5 Translational implications for cancer immunotherapy

Several approaches could, in principle, modify different parts of the framework discussed above. The three approaches discussed here—β-adrenergic blockade, psychosocial intervention, and serotonergic modulation—act through different mechanisms and should not be considered interchangeable. None has been established in a randomized trial as a strategy to improve ICI efficacy.

### 5.1 β-adrenergic blockade combined with ICIs

Preclinical studies show that β-adrenergic blockade can improve CD8^+^ T-cell function and enhance checkpoint blockade in selected models (32, 33, 35). Clinical evidence remains limited and inconsistent. The most direct datum is a phase 1 dose-escalation study of propranolol with pembrolizumab in treatment-naive metastatic melanoma: across nine patients, there were no dose-limiting toxicities, and propranolol 30 mg twice daily was selected as the recommended phase 2 dose (42). The study was designed to assess safety and dosing and cannot establish efficacy. Retrospective analyses of patients receiving β-blockers concurrently with ICI therapy have yielded mixed results, with some studies reporting improved disease control or progression-free survival, particularly in urothelial carcinoma and NSCLC (43, 44), but all are vulnerable to confounding in both directions, since β-blocker users differ systematically in cardiovascular comorbidity and concomitant medication. β-blockers also differ pharmacologically. Atenolol is predominantly β1-selective, whereas propranolol is non-selective, and pooled observational analyses rarely distinguish receptor-specific effects. β-adrenergic receptors are also expressed on tumour and stromal cells, and earlier studies describe effects on angiogenesis, invasion, and other non-T-cell components of tumour biology (11, 29–31). A clinical β-blocker association therefore cannot automatically be attributed to restored T-cell immunity.

Meta-analyses have not resolved the question. A pooled analysis across solid tumours found no significant association between β-blocker use and overall or progression-free survival in ICI-treated patients (45), and a more recent systematic review and meta-analysis of 12 studies and 4293 patients likewise found that adding β-blockers to ICIs was not associated with longer survival (46). At present, β-blockade should not be considered an evidence-based adjunct to ICI therapy. Any effect may depend on tumour type, receptor subtype, drug selectivity, and clinical context. Randomized biomarker-informed studies are needed before this approach can be considered clinically actionable. An ongoing single-centre phase 1 study is evaluating propranolol with sintilimab and platinum-based chemotherapy as first-line treatment for advanced NSCLC (BRIO; NCT05979818; estimated enrolment 6). No results are currently posted. The exercise literature—where acute adrenergic signalling is immunostimulatory (41)—is a further reason for caution about unselected β-blockade.

### 5.2 Psychosocial intervention

Psychological treatments reliably reduce anxiety and depression and improve quality of life in oncology populations: meta-analyses support small-to-moderate benefits of psycho-oncological intervention generally (47), of cognitive behavioural therapy (48), and of mindfulness-based interventions (49) on distress and related outcomes. A meta-analysis in breast cancer reported reductions in cortisol after psychosocial intervention (50). Whether such changes influence ICI efficacy is unknown. What is missing is outcome data. No adequately powered randomized trial has tested whether psychosocial intervention improves ICI efficacy as measured by progression-free or OS, and the optimal timing, intensity, and content of such intervention during ICI therapy are undefined. Psychosocial care is justified on quality-of-life grounds independently of any effect on tumour outcome (47). Whether it also improves checkpoint-blockade efficacy remains untested, and these two claims should not be presented as equivalent.

### 5.3 Serotonergic modulation and antidepressants

Antidepressants require separate consideration because their clinical indication differs from β-blockade and their immune effects are still being defined. Any clinical association is also vulnerable to confounding by indication. Experimental studies indicate that serotonergic pathways can influence antitumour immunity: the serotonin transporter (SERT) is induced on tumour-infiltrating CD8^+^ T cells and functions as a negative-feedback brake on their autocrine serotonin signalling, and its inhibition by selective serotonin-reuptake inhibitors (SSRIs) suppressed tumour growth, enhanced CD8^+^ effector responses, and synergized with PD-1 blockade across syngeneic and xenograft models, with intratumoral SERT expression inversely associated with survival across human cancers (51). A complementary mechanism has been described whereby the SSRI citalopram modulates complement C5a receptor 1 (C5aR1)^+^ tumour-associated macrophages in hepatocellular carcinoma (52). These findings should not be generalized to all antidepressants. SSRIs differ in pharmacological selectivity, dose, off-target effects, and clinical indication, and an immune effect observed with one compound may not apply to another. Notably, this effect appears at least partly independent of the serotonin transporter and should not be read as a purely serotonergic pathway—a reminder that the antitumour effects of SSRIs may operate through more than one route. Major cytochrome P450-mediated pharmacokinetic interactions with ICIs are not expected because monoclonal antibodies are not cytochrome P450 substrates. This does not exclude pharmacodynamic or immune interactions. Nonetheless, the clinical question—whether antidepressant use modifies ICI efficacy in patients—remains unresolved and is confounded by the depression for which these agents are prescribed; the appropriate principle is that depression should be treated on its own merits, while any effect on ICI outcome is regarded as a hypothesis for prospective study rather than a basis for practice.

### 5.4 Toward an integrative, evidence-graded model

These intervention strategies address different parts of the proposed framework: psychosocial care targets the clinical burden of distress, whereas β-blockers and serotonergic approaches target specific biological pathways. The strength of this model is that its first component, screening and psychosocial support, is justified independently of any effect on tumour outcome (47); its limitation is that the more ambitious claim—that modifying distress improves ICI efficacy—rests on association and mechanism rather than on interventional proof. Neuroendocrine-directed pharmacological strategies should remain investigational until randomized clinical data are available.

## 6 Limitations of the evidence and future directions

### 6.1 Association, not causation

The main limitation is that the clinical evidence is observational and cannot establish causation. Reverse causation remains possible because more advanced or symptomatic disease may increase psychological distress while independently worsening survival. Observational corticosteroid studies illustrate the related problem of confounding by indication. Baseline corticosteroids at 10 mg prednisone-equivalent or more at the start of PD-(L)1 blockade were associated with inferior response, progression-free survival, and OS in NSCLC (53). A subsequent analysis showed that this association was concentrated among patients receiving corticosteroids for palliative cancer-related indications such as dyspnoea and brain metastases, with no detriment evident among those treated for cancer-unrelated indications such as autoimmune disease (54). This illustrates how an exposure may mark a poor-prognosis subgroup rather than act as the direct cause of the observed outcome. Exogenous corticosteroid exposure is not equivalent to distress-related endogenous HPA-axis activation, and we do not treat the former as mechanistic evidence for the latter. Residual confounding by performance status, comorbidity, inflammatory burden, socioeconomic factors, sleep, physical activity, and treatment adherence is difficult to exclude observationally. The IPD analysis found adverse associations in both ICI and chemotherapy cohorts (20), but a formal treatment-by-distress interaction was not reported. Substantial preclinical evidence supports biological plausibility, but clinical causality has not been established. Current clinical evidence therefore more strongly supports a general prognostic association, while an ICI-specific predictive effect remains unproven. These findings argue against a simple unidirectional ICI-specific model.

### 6.2 Measurement and dynamics

Studies have used different instruments and thresholds, limiting direct comparison. A PHQ-9 or GAD-7 threshold is not equivalent to a cut-off on an EORTC emotional-functioning scale or to a recorded psychiatric diagnosis, and even PHQ-9/GAD-7 composite definitions differ across studies. Most studies have also assessed distress once, at baseline, neglecting its dynamic course. Distress may spike at initiation and settle with adaptation, and these trajectories may be more informative than a single measurement; high-frequency longitudinal monitoring, potentially via digital ecological momentary assessment, would allow the timing of distress to be related to immunological events and to the windows in which intervention might matter most. Host physiology also changes over time. Outside oncology, matched skin-barrier injury produced different systemic IL-33 responses across the circadian cycle, with skin-surface pH acting as a gate (55). This example shows why the timing of host-state and biomarker measurements may matter biologically. In an exploratory longitudinal analysis of STRESS-LUNG, ORRs were 65.3%, 57.1%, 69.2%, and 36.1% in the never, onset, remission, and persistence groups, respectively (18). Compared with persistent distress, distress remission was associated with a higher ORR (69.2% vs 36.1%; p=0.004) and longer PFS (12.7 vs 6.5 months; HR 0.51, 95% CI 0.27–0.94; p=0.030), whereas OS did not differ significantly (HR 0.69, 95% CI 0.30–1.61; p=0.394). Because remission was not randomized, these findings should be interpreted as exploratory longitudinal associations rather than evidence that distress reduction improves ICI efficacy. The reverse direction is also plausible. Tumour response, symptom relief, or better general health may reduce distress. Future studies should report the instrument, threshold, timing, and longitudinal change explicitly.

### 6.3 From mechanism to patient

The mechanistic literature rests heavily on murine models—chronic-restraint and housing-temperature paradigms (34, 35)—whose correspondence to chronic human psychological stress is imperfect, and the ADRB1/ADRB2 question (32, 33) remains unresolved in human tumours. Most experimental studies manipulate adrenergic and glucocorticoid pathways separately, so their combined contribution to checkpoint response remains uncertain. Circulating markers such as noradrenaline, cortisol, and salivary α-amylase correlate inconsistently with the intratumoral immune state, and direct intratumoral measurement is invasive and variable. Integrated analyses of clinical samples may help identify biologically relevant activation of adrenergic or glucocorticoid pathways (37, 39). Such biomarkers should be validated before they are used to select patients for neuroendocrine-directed intervention.

### 6.4 Designing the necessary trials

The causal question requires randomized trials with a clearly defined baseline distress phenotype, repeated psychological assessment during treatment, and prespecified clinical and biological endpoints. Time of day should also be considered. Clinical studies have linked ICI infusion timing with outcome, but the evidence is mainly observational (56–58). Future trials should prespecify or record the timing of biomarker collection and treatment administration. Such trials should prespecify the neuroendocrine hypothesis—adrenergic, glucocorticoid, or both—incorporate biomarker stratification because effects may vary by tumour type, β-adrenergic receptor subtype, and drug selectivity (45, 46), and account for behavioural and contextual factors that accompany distress (41, 59). When the aim is to evaluate predictive rather than purely prognostic value, a treatment-by-distress interaction should be prespecified and reported with its confidence interval, whether or not it reaches significance. A well-powered null treatment-by-distress interaction would argue against an ICI-specific predictive role. They should attend to feasibility, adherence, and cultural variation in the uptake of psychological treatment, and may reasonably combine modalities within factorial or platform designs. Until these data exist, neuroendocrine-directed intervention to improve ICI efficacy remains investigational. Box 2 summarizes the design priorities for biomarker-informed trials aimed at testing causality.

## 7 Conclusion

Psychological distress is a common and clinically consequential burden among patients with cancer treated with immune checkpoint blockade. Available evidence associates pretreatment distress with poorer outcomes in most, though not all, cohorts, and more strongly supports a general prognostic association. An ICI-specific predictive effect has not been demonstrated. β-adrenergic and glucocorticoid pathways provide plausible neuroendocrine links to impaired antitumour immunity, yet no randomized trial has shown that distress reduction improves immunotherapy efficacy.

Distress screening and appropriate psychosocial care should be part of supportive cancer care because distress is common, clinically important, and affects patient well-being. Future research should pair longitudinal distress assessment with neuroendocrine and immune biomarkers and use randomized, biomarker-informed designs to separate prognostic association from causal effects. Such care should not currently be presented as a strategy to improve ICI response or survival, and distress should not be regarded as a proven therapeutic target for improving ICI efficacy.

## Glossary

ACTH: adrenocorticotropic hormone
ADRB1: β1-adrenergic receptor
ADRB2: β2-adrenergic receptor
C5aR1: complement C5a receptor 1
cAMP: cyclic adenosine monophosphate
CI: confidence interval
CREM: cAMP-responsive element modulator
CRH: corticotropin-releasing hormone
CRP: C-reactive protein
CTLA-4: cytotoxic T-lymphocyte-associated protein 4
DCR: disease-control rate
DMFS: distant-metastasis-free survival
ECOG PS: Eastern Cooperative Oncology Group performance status
EORTC: European Organisation for Research and Treatment of Cancer
GAD-7: Generalised Anxiety Disorder-7
GILZ: glucocorticoid-induced leucine zipper
HPA: hypothalamic–pituitary–adrenal
HR: hazard ratio
ICI: immune checkpoint inhibitor
IFN: interferon
IFN-γ: interferon-γ
IL-6: interleukin-6
IL-10: interleukin-10
IPD: individual patient data
LAG-3: lymphocyte-activation gene 3
MDSC: myeloid-derived suppressor cell
MHC: major histocompatibility complex
MPR: major pathological response
NK: natural killer
NR3C1: nuclear receptor subfamily 3 group C member 1 (glucocorticoid receptor)
NSCLC: non-small-cell lung cancer
ORR: objective response rate
OS: overall survival
PD-1: programmed cell death protein 1
PD-L1: programmed death-ligand 1
PFS: progression-free survival
PHQ-9: Patient Health Questionnaire-9
PRISMA: Preferred Reporting Items for Systematic Reviews and Meta-Analyses
PSM: propensity-score matching
QoL: quality of life
RFS: relapse-free survival
SERT: serotonin transporter
SSRI: selective serotonin-reuptake inhibitor
TIGIT: T-cell immunoreceptor with immunoglobulin and ITIM domains
TIL: tumour-infiltrating lymphocyte
TIM-3: T-cell immunoglobulin and mucin-domain containing-3
TMB: tumour mutational burden
TME: tumour microenvironment
Treg: regulatory T cell
TTF: time to treatment failure.
